# Upward violence against nurse managers in the workplace: a scoping review of prevalence, influencing factors and coping strategies

**DOI:** 10.3389/fpubh.2025.1681405

**Published:** 2025-12-11

**Authors:** Zhiwei Xu, Li Li, Xiaomei Gan, Wenfang Mu, Ying Wu

**Affiliations:** 1 Department of Clinical Research Center, Shanghai Mental Health Center, Shanghai Jiao Tong University School of Medicine, Shanghai, China; 2 Department of Nursing, Shanghai Mental Health Center, Shanghai Jiao Tong University School of Medicine, Shanghai, China; 3 Department of Nursing, Renji Hospital, Shanghai Jiao Tong University School of Medicine, Shanghai, China

**Keywords:** nurse manager, upward violence, workplace violence, workplace bullying, workplace incivility

## Abstract

**Background:**

Although workplace violence is a well-documented concern in healthcare, most research has focused on vertical and horizontal forms, with limited attention to upward violence. This overlooked phenomenon may undermine nurse managers' occupational well-being and diminish their organizational commitment. To address this gap, this scoping review synthesizes and maps the existing evidence on upward violence against nurse managers.

**Methods:**

We searched relevant literature available in such academic databases as MEDLINE, Embase, CINAHL, APA PsycINFO, Cochrane Library, PubMed and Web of Science. The retrieval time limit was from the establishment of the database to March 2025.

**Results:**

A total of 9 articles were included, among which there were 5 quantitative studies, 3 qualitative studies and 1 mixed-methods study. The nurse managers are facing upward violence in the workplace. Its occurrence is affected by leadership styles, role overload, organizational restructuring, hostile emotions, and resource allocation, and it will cause harm to the managers in terms of physical, psychological, financial, work quality and career development. Confrontation, critical conversations, silence, incident reporting, environmental support and leaving or transferring departments are the main coping styles of nurse managers in this review.

**Conclusion:**

In the future, high-level nursing managers should pay more attention to the problem of upward violence, and at the same time strengthen the manager's awareness of reporting and formulate corresponding intervention strategies according to its characteristics to reduce its negative impact.

**Systematic review registration:**

https://osf.io/cq6vp.

## 1 Introduction

Workplace violence in healthcare settings has become a significant occupational hazard worldwide, with nurses often being primary victims (1). Previous study have reported that nurse managers are exposed to workplace violence at a prevalence rate as high as 60% (2). While research in the nursing field has extensively explored vertical violence (from superiors to subordinates) and horizontal violence (between peers), the reverse direction of violence has received comparatively less attention. As central figures in nursing management, nurse managers may, under certain circumstances, become targets of violent behaviors initiated by their subordinates, a phenomenon known as upward violence. This type of violence differs from conventional forms of workplace aggression and has often been obscured by the predominant focus on vertical and horizontal violence, leading to its frequent oversight and underreporting.

Upward violence refers to violent acts committed by subordinate individuals against authority figures within organizational hierarchies (3). This concept was initially introduced by Miller (4) in a discussion of gender-based harassment and later explored in preliminary studies within nursing contexts by Branch et al. (5). As frontline administrators and clinical practitioners, nurse managers play a vital role in ensuring nursing care quality. Studies reveal that 20.2% of nurse managers have been identified as targets of upward violence in the workplace. Such violence not only undermines nurse managers' leadership effectiveness but also causes equivalent harm to that caused by other forms of violence (2, 6, 7).

While the phenomenon of upward violence in nursing is gradually receiving academic attention, Parsons et al. (8) have summarized the impact of upward violence on nursing managers; however, a comprehensive review examining its prevalence, contributing factors, and coping strategies remains absent. Scoping reviews serve as efficient tools to summarize research progress, synthesize current findings, and pinpoint knowledge gaps within specific domains. Consequently, this study employs scoping review methodology to systematically search, screen, and synthesize existing research on nurse managers' experiences with upward workplace violence. The objectives are to thoroughly map the current research landscape and provide foundational references for future investigations.

Additionally, in research on workplace violence, terms such as bullying, mobbing, abuse, aggression, and incivility are often used interchangeably (9). Many studies employ the term “violence” as an umbrella concept to encompass these negative behaviors. Regardless of their specific forms, this review focuses on upward violence within the nursing context. Therefore, we use the term “violence” to encompass all types of negative workplace behaviors.

## 2 Methods

This scoping review was conducted using the Joanna Briggs Institute (JBI) methodology and reported in accordance with the Preferred Reporting Items for Systematic Reviews and Meta-Analyses extension for Scoping Reviews (PRISMA-ScR) statement (10, 11). The project was registered at OSF (https://osf.io/cq6vp, registration doi: 10.17605/OSF.IO/CQ6VP).

### 2.1 Research questions

This scoping review explored the issue of upward violence experienced by nurse managers in the workplace. The specific questions addressed were:

- What are the prevalence and influencing factors of upward violence against nurse managers in the workplace?
- What are the negative impacts of upward workplace violence on nurse managers?
- What coping strategies do nurse managers employ when facing upward violence?

### 2.2 Inclusion and exclusion criteria

The inclusion criteria were developed using the PCC (Participants, Concept, Context) framework (12): (a) Participants: Nurse managers (including head nurses, nursing supervisors, or equivalent administrative roles); (b) Concept: In studies on upward violence, nurse managers are identified as the victims of workplace violence while simultaneously holding a position of authority over the nurses who direct violence toward them; (c) Context: Healthcare settings (e.g., hospitals, clinics, long-term care facilities, community health centers).

Exclusion criteria: (a) non-English publications; (b) unavailable full texts; (c) secondary literature types: reviews, conference abstracts, research protocols, editorial letters, book reviews, and news reports.

### 2.3 Search strategy

The following databases were systematically searched: MEDLINE, Embase, CINAHL Complete, APA PsycINFO, Cochrane Library, PubMed, and Web of Science. Search strategy included combinations of keywords and Boolean operators. Table 1 displays the detailed search strategy applied in PubMed and CINAHL. The final search update was performed in March 2025. Meanwhile, the reference lists of the included studies were hand-searched to ensure that all relevant studies were identified and included. Keywords included: (a) “nurse administrators”, “nurse managers”, “charge nurses”, “head nurses”, “nursing management”, “nurs^*^ manag^*^”, “nurs^*^ administrat^*^”, “nurs^*^ leader^*^”, “senior nurse”, “senior nurs^*^”; (b) “upward violence”, “upwards bullying”, “workplace violence”, “aggression”, “bullying”, “verbal abuse”, “incivility”, “bullied”, “abuse”, “mobbing”, “hostil^*^”, “violence”.

**Table 1:** Search strategy.

**Database**	**Search string**
**PubMed**	#1 (Nurse Administrators [MeSh]) OR (Head Nurses [MeSh])
	#2 (Nurse Managers [Title/Abstract]) OR (Charge Nurses [Title/Abstract]) OR (Nursing Management [Title/Abstract]) OR (Nurs* Manag*[Title/Abstract]) OR (Nurs* Administrat*[Title/Abstract]) OR (Nurs^*^ Leader^*^[Title/Abstract]) OR (Senior Nurse[Title/Abstract]) OR (Senior Nurs^*^[Title/Abstract])
	#3 #1 OR #2
	#4 (Upwards Bullying [MeSH]) OR (Bullying [MeSH]) OR (Aggression [MeSH]) OR (Verbal Abuse [MeSH]) OR (Incivility [MeSh]) OR (Workplace Violence [MeSH])
	#5 (Bullied [Title/Abstract]) OR (Abuse [Title/Abstract]) OR (Violence [Title/Abstract]) OR (Mobbing [Title/Abstract]) OR (Hostil*[Title/Abstract])
	#6 #4 OR #5
	#7 #3 AND #6
**CINAHL**	#1 MH “Nurse Administrators” OR MH “Head Nurses” OR MH “Nurse Managers” OR MH “Charge Nurses” OR MH “Nursing Management” OR XB (Nurs* Manag*) OR XB (Nurs* Administrat*) OR XB (Nurs^*^ Leader^*^) OR XB (Senior Nurse) OR XB (Senior Nurs^*^)
	#2 MH “Bullying” OR MH “Aggression” OR MH “Verbal Abuse” OR MH “Incivility” OR MH “Workplace Violence” OR “Violence” OR XB (Bullied) OR XB (Abuse) OR XB (Mobbing) OR XB (Hostil*)
	#3 #1 AND #2

### 2.4 Study selection

All retrieved records were imported into EndNote for duplicate removal. Two authors independently conducted primary screening by reviewing titles and abstracts against the eligibility criteria. Potentially eligible studies subsequently underwent full-text screening. Discrepancies were resolved through discussion with a third author until consensus was reached on the final inclusions.

### 2.5 Data extraction

Data extraction was performed independently by two authors with systematic training in evidence-based nursing. Discrepancies were reconciled through consultation with a third author. Extracted information included: (a) Country and publication year; (b) Research focus/themes; (c) Sample characteristics and study design; (d) Key findings relevant to the research questions. All three authors iteratively reviewed and analyzed the included literature to reach a consensus on data synthesis.

## 3 Results

**Figure 1:**
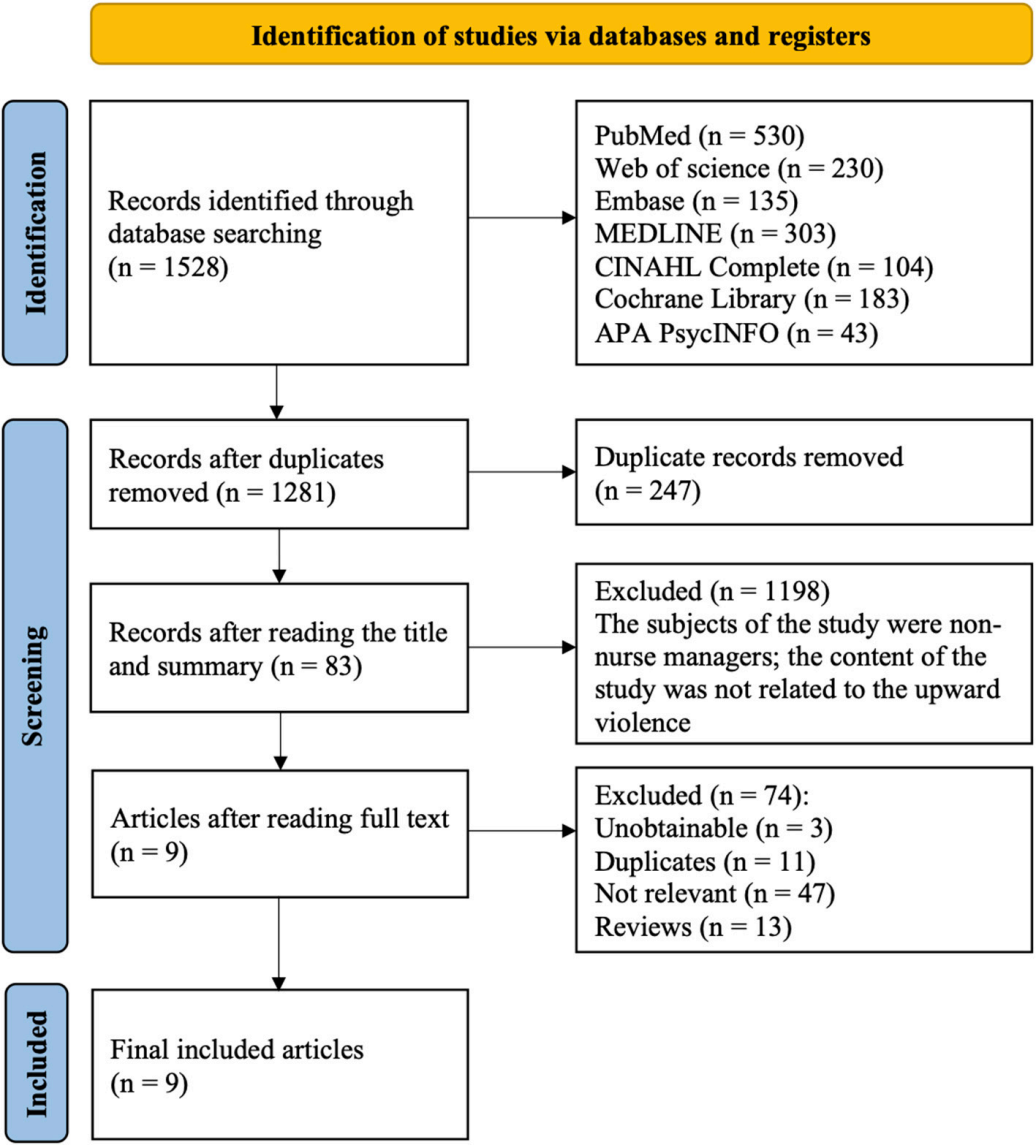
PRISMA flowchart of study selection.

The initial database search identified 1,528 records. After removing 247 duplicates, 1,281 records remained. Screening of titles and abstracts excluded 1,198 irrelevant studies due to mismatched populations or non-relevant topics. Full-text review of the remaining articles led to the exclusion of 74 articles, including 3 with unavailable full texts, 11 duplicate studies, 47 irrelevant research topics, and 13 review articles. Ultimately, 9 articles were included for analysis (2, 7, 13–19), comprising 5 quantitative studies (2, 7, 13, 15, 17), 3 qualitative studies (14, 16, 19), and 1 mixed-methods study (Figure 1) (18). The characteristics of included articles are summarized in Table 2.

**Table 2:** Summary of selected articles.

**Authors, Year, Country**	**Aims**	**Concept definition**	**Study design**	**Data collection instruments**	**Time frame**	**Sample information (participants, recruitment methods, sample size)**	**Main results**
Parchment et al. (7), 2019, USA	Investigate the incidence of workplace bullying and related environmental factors among nurse managers	Workplace bullying is recurring, intimidating, and offensive verbal and nonverbal behavior that damages the physical and emotional well-being of individuals	Quantitative study	The Negative Acts Questionnaire–Revised (NAQ-R), The Upwards Bullying Scale, The Authentic Leadership Inventory, The Global Social Power, The Health and Safety Executive Management Standards Work-Related Stress Indicator Tool (HSEMS IT)(only the job demand and job control subscales were utilized)	Over an entire career	A large national nursing leadership organization as well as a small regional nursing leadership organization with combined membership databases of ~9,500 members was used to recruit nurse managers. A total of 241 nurse managers completed the survey.	Nurse managers as recipients of workplace bullying predominately from their nurse leaders, subordinate clinical nurses, and fellow nurse managers. Leadership styles have been identified as major contributing factors to workplace violence.
Karsavuran et al. (13), 2015, Turkey	Analyze the relationship between burnout and mobbing among hospital managers	Mobbing refers to the repeated behavior of individuals or groups that intentionally harms others with whom they work	Quantitative study	The Leymann Inventory of Psychological Terrorization (LIPT)	Over an entire career	A convenience sampling method was employed to survey all 454 managers at the hospitals. Of these, 54% (244 managers) responded to the questionnaire.	Head nurses who experience mobbing and burnout are more likely to deliver suboptimal care, which may contribute to adverse outcomes.
Hampton et al. (2), 2018, USA	Investigate the experience of nursing leaders in coping with workplace bullying	Workplace bullying refers to the persistent exposure to negative acts that are intended to humiliate, offend, undermine, degrade, ridicule, and induce stress	Quantitative study	Revised Negative Acts Questionnaire (NAQ-R)	Over an entire career	Participants were recruited for the study through an announcement about the study on the leadership organization's web page, which included 175 nurse managers, directors and nursing executives.	Bullying is a significant workplace stressor for nurse managers, and there are no simple solutions to this serious issue within the healthcare work environment.
Tuna et al. (14), 2019, Turkey	Determine the causes of workplace bullying and its impact on nurse managers	Workplace bullying is defined as repeated hostile behaviors by a person or group to another person	Qualitative study	Semi-structured interviews: “(a) Have you ever been exposed to or were you exposed to violent behaviors by your subordinates/other nurses such as pressure, intimidation and humiliation? (b) If you have been exposed to such violent behaviors, why do you think that they have happened? (c) If you have been exposed to such violent behaviors, by whom have you been exposed to? Is it a nurse or a group of nurses? (d) Have you ever been exposed to violent behaviors by other nurse managers-other nurses in charge such as humiliation and insult? If yes, by whom and how have you been exposed to? (e) Have you ever been exposed to violent behaviors by your manager or nurse manager such as humiliation and insult? If yes, how have you been exposed? (f) How do you react when you are exposed to violent attitudes and behaviors by a nurse or a group of nurses? (g) What kind of coping strategies do you apply when you are exposed to violence by your subordinates/other nurses? (h) What kind of coping strategies do you apply when you are exposed to violence by your manager or nurse managers? (i) What kind of changes have these violent and humiliating behaviors brought in your perspective of your workplace/working environment? (j) What kind of changes have these violent and humiliating behaviors brought in your personality?”	Over an entire career	A convenience sampling method was employed to conduct the interviews, involving 25 nurses who had a minimum of two years of experience as hospital ward nurse managers and who led inpatient units.	Nurse managers are exposed to bullying within their units, which directly affects their performance and has both positive and negative impacts on them.
Tarasenko et al. (15), 2022, USA	Determine the relationship between practice environment variables and nurse manager perceptions of workplace mistreatment	Workplace mistreatment encompasses uncivil, socially deviant, bullying, and tyrannical behaviors	Quantitative study	The Workplace Mistreatment Questionnaire (WPMQ), Four subscales from the Nurse Manager Practice Environment Scale (NMPES) (collegial physician relationships; constructive director relationships; fair and manageable workload; adequate budgeted resources)	Over an entire career	A total of 139 participants were recruited as a convenience sample of nurse managers through internal and external advertisements from a national nursing leadership organization, ambulatory care facilities, state boards, and a nursing association.	Mistreatment is prevalent in nurse managers' practice environments and provided explanations regarding the relationship between budgeted resources and such incidents.
Lasala et al. (16), 2016, USA	Understand the consequences of incivility and bullying experienced by nursing academic administrators	Bullying, is more than the disrespect of incivility because it is intentional and focuses on particular individuals or groups	Qualitative study	Semi-structured interviews: Each interview began with an open-ended statement of “Tell me about your experience with incivility and bullying in the workplace aimed at you in your nursing administrative role.”	Over an entire career	A purposive sample of 14 administrators from nationally accredited nursing programs was obtained through an emailed letter utilizing snowball sampling.	Findings revealed that incivility had devastating effects on administrators personally and professionally.
Harlos et al. (17), 2005, Canada	Investigate the nature of workplace mistreatment experienced by hospital managers	Mistreatment can be defined as behaviors that employees perceive as unjust or abusive	Quantitative study	Developed content-valid scales to measure workplace mistreatment using items drawn from previous research. A total of 41 items were generated, comprising 12 positive items and 29 negative items	Previous 12 months	Using a convenience sampling method, 125 managers from a hospital in Western Canada were randomly selected.	The study suggests that: (a) mistreatment experiences can be differentiated based on whether they are interpersonal or organizational in nature; (b) organizational mistreatment is multidimensional; and (c) contextual support serves as a mediating mechanism between work mistreatment and both work attitudes and employees' psychological well-being.
Andersen et al. (18), 2019, Australia, USA	To determine the types and frequency of incivility and unprofessional nursing student behaviors in the clinical setting	Incivility refers to rude or disruptive behavior that often causes distress for all parties involved, including those who are directly targeted, observers, and organizations	Quantitative and qualitative study	Difficult Nursing Student Behaviors and Teaching Situations (DNBTS); Semi-structured interviews: “Your experiences about student incivility and unprofessional behaviors.”	Previous 12 months	A cross-sectional purposeful sample of 71 clinical nursing educators was recruited from two Schools of Nursing within Australia and one in the United States.	Almost all participants had experienced nursing students' incivility and unprofessional behaviors. Clinical educators with formal training and experience in education reported a higher number and frequency of such incidents.
He et al. (19), 2024, China	Understand the current situation of upward bullying and explore the manifestations, reasons, and outcomes of it	It is characterized by repeated intimidating, aggressive, and hostile behavior that includes name-calling, harassment, humiliation, cold aggression, and the devaluation of job results	Qualitative study	Semi-structured interviews: “(a) Have you heard of the term “upward bullying”? What do you understand by “upward bullying”? (b) Have you ever been the victim of upward bullying or witnessed it among others since becoming a nurse manager? (c) If you have been exposed to upward bullying, what did you specifically witness or experience? (d) If you have been the victim of upward bullying, why do you think it occurred? (e) How has upward bullying in the workplace affected you? How do you feel about it? (f) What measures are in hand to help reduce the incidence of upward bullying in your workplace?”	Over an entire career	Purposive sampling was used to recruit 12 nurse managers from two tertiary general hospitals and one secondary general hospital.	Nurse managers are exposed to upward bullying in various forms and due to a range of complex factors. Greater emphasis should be placed on fostering a positive work environment for them to facilitate their managerial role.

### 3.1 Characteristics of included studies

A total of nine studies published between 2005 and 2024 were included, with the highest number of studies published in 2019 (3/9, 33.3%). The majority of the studies originated from the United States (44.4%, *n* = 4), followed by Turkey (22.2%, *n* = 2), and one study each from Canada, China, and Australia (each accounting for 11.1%), with the Australian study being a multicenter study. According to Table 2, the literature included primarily consists of quantitative research (55.6%, *n* = 5), followed by qualitative research (33.3%, *n* = 3) and mixed-method research (11.1%, *n* = 1). Among the five quantitative studies included, all were cross-sectional observational designs with relatively large sample sizes, all having *n* ≥ 120. Of the qualitative studies, two explicitly reported using a phenomenological approach, while the remaining two, including one mixed-methods study, did not specify their qualitative paradigm.

### 3.2 Prevalence of upward violence against nurse managers

Five quantitative studies and one mixed-method study consistently report that managers frequently experience upward violence from nursing staff. This finding emphasizes that such workplace violence constitutes persistent patterns of aggression rather than isolated incidents, with escalating severity over time (2, 7, 13, 15, 17, 18). Additionally, four cross-sectional studies and one mixed-method study reported the occurrence of upward violence, with prevalence rates ranging from 19.7% to 61.1% among nurse managers (2, 7, 15, 18). Notably, Hampton et al. (2) identified that nearly 26% of nurse managers experienced severe workplace bullying. Karsavuran and Kaya (13) revealed that collective bullying targeting nurse managers also occurs within healthcare settings. Similarly, Lasala et al. (16) demonstrated that academic nursing administrators faced covert hostility (e.g., disrespectful behaviors) from nursing faculty members. Furthermore, Andersen et al. (18) identified a progressive increase in the frequency of upward violence perpetrated by nursing students during clinical education placements. All nine included articles highlighted nurse managers expressed need for support. However, due to leadership style variations and the covert nature of abusive behaviors (e.g., sarcasm, ostracism, fabricated accusations), some managers were more inclined to remain silent.

### 3.3 Influencing factors of upward violence against nurse managers

#### 3.3.1 Leadership styles

Four cross-sectional studies highlighted the relationship between leadership styles and upward violence (7, 13, 15, 17). Nurse managers adopting authentic leadership styles exhibited lower rates of violence reporting. In contrast, rigid leadership styles combined with limited control over job responsibilities were associated with increased incidents of upward violence.

#### 3.3.2 Role overload

Qualitative studies by Tuna and Kahraman (14) and He et al. (19) indicated that role overload significantly increased nurse managers' vulnerability to aggression from subordinates. This occurs when managers assign excessive responsibilities to staff, escalating work-related stress and redirecting dissatisfaction toward leadership. In the study by Tuna and Kahraman (14), one nurse manager described this experience: “Because nurses have less tolerance due to reasons especially like lack of personnel, workload, and intensive watches and I am their closest manager, they may directly reflect their negative emotions on me.”

#### 3.3.3 Organizational restructuring

Three cross-sectional studies demonstrated that nurse managers frequently experienced upward violence during organizational changes (2, 7, 15), such as personnel adjustments, departmental mergers, or unit reorganizations.

#### 3.3.4 Hostile emotions

Three qualitative studies have linked hostile emotions within social groups—including resentment, hostility, jealousy, and prejudice—to upward violence against nurse managers (14, 16, 19). Specific triggers included conflicts over clinical workload distribution, educational disparities, managerial seniority, and gender-related biases.

#### 3.3.5 Resource allocation

In a quantitative analysis, Tarasenko et al. (15) identified limited budgetary resources as a key predictor of violence. During resource reallocation, 61.1% of nurse managers reported encountering passive resistance, uncivil emails, threats, derogatory remarks, and gossip from nursing staff. Similarly, in the qualitative study by He et al. (19), a newly appointed manager stated: “The new head nurse is bound to change some of the management mode. And the nurses who have taken the ‘bonus' of the previous manager will have a gap in their hearts, thinking that their benefits have changed from more to less.”

### 3.4 Negative impacts of workplace upward violence on nurse managers

The results indicate that upward violence causes physical, psychological, financial, and professional damage to nurse managers. Three qualitative studies revealed a range of consequences from diminished self-esteem to serious health outcomes, including hypertension, weight loss, and post-traumatic stress disorder (PTSD) (14, 16, 19). Furthermore, three cross-sectional studies documented that behaviors such as humiliation, threats, disrespect, and social isolation triggered psychological symptoms, including anxiety, tension, depression, accompanied by insomnia and appetite disturbances (2, 13, 15). Studies by He et al. (19) and Lasala et al. (16) also highlighted the economic burdens borne by nurse managers to mitigate violence impacts, including legal fees and healthcare expenditures, alongside a decline in nursing management quality. Chronic exposure to bullying environments reduced organizational commitment and increased career burnout, with four studies reporting elevated turnover intention among victimized nurse managers (2, 7, 13, 14). Notably, He et al. (19) also identified positive outcomes arising from violence, such as self-reflection and personal growth. One nurse manager who participated in the study shared: “Management work is not infallible, all things have room for improvement. Why people are sometimes in a bad mood is something I need to think about, and I will take the time to work on myself.”

### 3.5 Coping strategies of nurse managers against upward workplace violence

The primary coping strategies identified among nurse managers included confrontation, critical conversations, silence, formal reporting, and ultimate career disengagement through resignation or unit transfer. According to Hampton et al. (2), ~40% of nurse managers adopted active response strategies (e.g., direct confrontation or dialogue with the aggressor) to mitigate violent behaviors. Studies by Hampton et al. (2) and Tuna and Kahraman (14) found that nearly half of nurse managers perceived silence or avoidance as the most effective short-term strategy to prevent escalation. However, prolonged exposure to workplace violence led 16% of nurse managers to resign or transfer due to deteriorating mental and physical health. Harlos and Axelrod (17) demonstrated that environmental support strategies enhanced psychological resilience, buffering against verbal abuse, work obstruction, and emotional neglect. Notably, only 5% of nurse managers escalated incidents to Human Resources or senior leadership (2).

## 4 Discussion

### 4.1 High prevalence of upward violence with predominant silence among nurse managers in healthcare settings

The review findings indicate a high prevalence of upward violence experienced by managers in workplace settings. Prevalence estimates exhibit significant variability across different study regions and assessment methodologies, ranging from 19.7% to 61.1%. This variability may be influenced by organizational silence. Studies have shown that 61.6% of nursing personnel chose to remain silent when confronted with critical issues (17, 20). Several factors contribute to the silence of nurse managers following incidents of workplace violence. From the perspective of organizational silence behavior theory, Pinder and Harlos (21) suggested that when an organization is characterized by a strong climate of silence, an unfair culture, limited communication channels, and a tendency to ignore employee feedback, silence is more likely to occur. Furthermore, as healthcare institutions are recognized as high-risk environments for workplace violence and bullying, such repeated exposure reinforces and perpetuates an organizational culture of silence within these settings.

As middle-level leaders, nurse managers report to nursing directors or higher-level administrators. Morrison and Milliken (22) noted that leaders often fear receiving negative feedback, particularly when such information originates from subordinates. Moreover, leaders may view subordinates as self-interested and untrustworthy, rather than as individuals genuinely committed to organizational goals. This perception can create discomfort for nurse managers when expressing their opinions, as they may fear that disclosing experiences of workplace violence could be interpreted as a sign of inadequate leadership. As a result, they may be reluctant to report or discuss such incidents, thereby further reinforcing a climate of organizational silence.

Finally, according to self-determination theory (23), individuals tend to engage in behaviors that promote personal and collective growth when their basic psychological needs are fulfilled within a supportive social environment. Conversely, when these needs are thwarted, individuals may resort to behaviors detrimental to themselves or others. Workplace violence can undermine nurse managers' sense of control over their environment, and when their fundamental psychological needs are not met, they may choose silence or other maladaptive coping responses that negatively affect both the individual and the organization.

Although remaining silent may help managers avoid direct confrontation with aggressors, study have confirmed that victims' passive and ineffective coping strategies may encourage further acts of workplace violence by the aggressors, which typically escalate over time and persist for at least 6 months (24). Such behavior not only undermines the leadership capacity of nurse managers but also negatively affects their organizational commitment (25). According to Milliken et al. (26), organizational silence can lead employees to experience a loss of control over their work. From the perspective of nurse managers, maintaining silence on issues within their sphere of competence often results in frustration, tension, and feelings of exclusion. Persistent silence may further cause them to question their value and legitimacy, leaving them in a vulnerable position where they are unable to effectively exercise their formal authority. As power imbalances and changes in power dynamics occur, these conditions may further exacerbate the incidence of upward violence.

While existing studies on horizontal violence and prevention of occupational burnout have provided valuable insights into reducing organizational silence, future research should extend these findings to enhance nurse managers' awareness and reporting of upward violence. Evidence suggests that scenario-based training, personal reflection, and peer support can strengthen nurses' voice behaviors and help mitigate organizational silence (27–29). Moreover, healthcare institutions should establish formal support and intervention mechanisms, including access to professional psychological or mental health services, grievance procedures, and union-based actions, to more effectively address the issue of upward bullying in the workplace.

### 4.2 Multiple contributing factors to upward workplace violence

This review identified leadership styles, role overload, organizational restructuring, hostile emotions, and resource allocation as key contributors to upward violence against nurse managers. Regarding leadership styles, a study indicated that ineffective authentic leadership among nurse managers may foster negative behaviors and attitudes among nurses, potentially escalating into detrimental tendencies (30). Furthermore, related research found that authentic leadership plays a moderating role in relationship conflicts. Although its level may vary among individuals, it can be developed and strengthened (31).

Concerning role overload, research by Hoel et al. (32) indicates that in practical work environments, managers are often required to assume responsibilities typically handled by specialized departments, such as Human Resources. If managers do not clearly define the boundaries of authority and responsibility, this can increase employees' workload and potentially make managers targets of violence. Our findings align with this perspective. Furthermore, the study suggests that females experience higher role overload compared to their male counterparts, likely due to societal norms that impose dual pressures: professional responsibilities and subjective obligations toward domestic duties, such as childcare and eldercare (33).

Organizational changes, including mergers and departmental realignments, further predict upward violence. Torkelson et al. (34) found that when restructuring triggers employee concerns about job stability or career prospects—or when staff perceive themselves as victims of such changes—managers may become targets of displaced frustration. This finding is consistent with the results of the present review. Drawing on social identity theory (24), managers may be perceived as representatives of the “out-group” during organizational transitions, making them vulnerable scapegoats for collective discontent.

Moreover, workplace violence may also arise when hostile emotions such as resentment, jealousy, and prejudice emerge within social groups. In such situations, high-achieving managers are often targeted (35). Related research suggests that managers may unknowingly become targets of upward violence when aggressors feel resentment over missed promotion opportunities or jealousy toward peers who have advanced into managerial roles (32).

Resource allocation constitutes another significant factor contributing to upward violence, as it directly shapes employee attitudes and behaviors. A study by Samnani and Singh (36) examined the role of performance-enhancing compensation practices in stimulating bullying behaviors and identified these practices as significant triggers of workplace violence. In typical circumstances, when the resource pool is adequate, top-performing employees may receive substantial rewards, while average or low-performing staff receive comparatively smaller raises. This strategy can motivate lower performers to strive for higher compensation. However, when resources are limited, “zero-sum” compensation systems often evolve into a binary model of “haves” vs. “have-nots”. This dynamic frequently breeds jealousy and fosters a negative work environment, ultimately leading to upward violence against managers under the guise of unfair resource allocation.

Therefore, future research should focus on the protective role of clearly defined job responsibilities in preventing upward violence. It is essential for nurse managers to adopt more positive leadership styles and facilitate effective communication with team members during organizational restructuring to ensure a clear and timely understanding of transitional processes. For instance, authentic leadership can be strengthened through targeted training programs or pre-employment onboarding initiatives. Baron (37) implemented an authentic leadership development program based on action learning principles, which significantly enhanced managers' authentic leadership capabilities. Furthermore, managers should prioritize the establishment of fair and equitable compensation systems to foster harmony and trust between nursing staff and the organization, thereby reducing the risk of violent incidents.

### 4.3 Severe impacts of upward violence on nurse managers' health and its association with turnover intentions

Chronic exposure to workplace violence imposes significant psychological stress on nurse managers, adversely impacting their physical and mental health, undermining the quality of nursing management, and reducing their organizational commitment. Labrague et al. (38) found that nurse managers, as frontline clinical administrators, generally exhibit suboptimal mental health status, with pronounced depressive symptoms that are further aggravated by exposure to workplace violence. According to the Conservation of Resources Theory (39), when individuals are exposed to ongoing stressors and are unable to prevent the depletion of psychological resources or obtain timely replenishment, they may enter a “loss spiral” of accelerated resource erosion. As these internal reserves approach exhaustion, individuals tend to adopt defensive strategies aimed at halting further loss, which may manifest as passive coping mechanisms, avoidance behaviors, and declining work performance (40). At the same time, severe psychological strain often necessitates medical interventions for stress-related conditions, resulting in considerable financial burden. Prolonged resource depletion without effective replenishment ultimately leads to emotional exhaustion, driving nurse managers to alleviate distress by resigning from their positions. As present, the nursing workforce continues to face a critical shortage, with the cost of replacing each nurse manager estimated at 75% to 125% of their annual salary (41, 42). The turnover of nurse managers thus imposes severe financial and operational burdens on healthcare institutions.

Given these consequences, future research should emphasize the enhancement of psychological capital among managers who encounter upward violence in the workplace, with the objective of mitigating their turnover intentions. This can be accomplished through a variety of interventions, including the Psychological Capital Intervention model, positive psychology interventions, stress management programs, and Job Demands-Resources interventions, all aimed at strengthening employees' psychological capital (43).

### 4.4 Cultural influences on upward violence in the workplace

The studies included in this review were primarily conducted in Western contexts, with a few originating from Turkey and China. Cultural differences, particularly the power distance inherent in various national cultures, appear to shape the direction of workplace violence. Concurrently, the reporting and expressions of violence exhibit certain cultural universals. According to Steers et al. (44), in cultures characterized by high power distance, authority tends to be concentrated at higher hierarchical levels, resulting in violence typically flowing downward. In contrast, low power distance cultures emphasize equality and a more dispersed distribution of power, allowing violence to flow not only downward but also horizontally or upward. This may help explain why the majority of studies included in this review were conducted in Western settings. Interestingly, a study by D'Cruz and Rayner (45). reported an upward violence prevalence of 21.8% in India—a country characterized by high power distance—an unexpectedly high figure. The investigation revealed that this phenomenon often resulted from cross-level co-bullying, where subordinates rarely initiated aggression independently but were supported by other managerial staff. Therefore, similar to workplace violence more broadly, upward violence may be shaped by cultural differences, which should be carefully considered when interpreting workplace violence across cultural contexts.

Secondly, a closer examination of the reporting and expressions of upward violence reveals certain underlying commonalities across cultures. In terms of expressions, the cultural context does not significantly alter the nature of workplace violence. Whether in Eastern or Western settings, its core characteristic lies in the persistent and repetitive mistreatment of a target over time, ultimately leading to a state of defenselessness. Regarding reporting behaviors, similar patterns are also observed across cultural contexts, most managers tend to remain silent after experiencing workplace violence. This finding aligns with the results reported by D'Cruz et al. (46), who found that among 114 participants from India (83%), Australia (64%), and Turkey (68%), the majority opted to remain silent after experiencing workplace violence. Although the specific reasons for underreporting may vary culturally, the underlying mechanisms of suppression are largely shared. These mechanisms may stem from both individual concerns and systemic factors, such as fears of losing anonymity or damaging one's reputation, worries about harming professional relationships, organizational cultures that discourage questioning or complaints, and the absence of formal grievance procedures.

### 4.5 Research on coping strategies for upward violence requires further enrichment

Although research on upward violence has gradually attracted the attention of scholars, this review identifies a significant gap in effective intervention strategies for nurse managers facing such aggression. Current coping approaches remain largely passive; however, ~40% of nurse managers adopt active response strategies (2), such as critical conversations, to mitigate workplace violence. Notably, this review found no relevant randomized controlled trials, resulting in a lack of high-quality evidence to validate the effectiveness of these active coping strategies. Moreover, related research (47) found that proactive engagement, such as direct communication with perpetrators, was effective only in cases of low-intensity violence. Importantly, Hutchinson and Hurley (48) demonstrated that managers with higher psychological resilience and emotional intelligence exhibited greater confidence when confronting aggression, which aligns with findings from He et al. (19). Therefore, future research should further explore these psychological traits as potential foundations for developing intervention strategies aimed at fostering a healthier work environment for nurse managers.

## 5 Conclusions

This review discloses that nurse managers face upward violence in workplace environments, influenced by factors such as leadership styles, role overload, organizational restructuring, hostile emotions, and resource allocation. Such violence causes multifaceted harm to nurse managers, affecting their physical and mental health, financial well-being, work performance, and career development. Notably, nearly half of nurse managers perceive silence or neglect as the most feasible coping strategy. Senior nursing administrators should prioritize raising awareness of upward violence, enhancing reporting mechanisms, and implementing targeted interventions to mitigate its negative impacts and foster healthier work environments. The limited number of included studies, predominantly from Western countries, highlights potential cultural biases and underreporting challenges due to the covert nature of upward violence.

## 6 Limitations

This study has several limitations. First, although a relatively comprehensive search strategy was employed, the number of eligible studies on upward violence in the nursing field remains limited. This is primarily due to the covert nature of upward violence and the difficulty in its identification. Second, most of the included studies relied on cross-sectional surveys or self-reported data, which may introduce self-reporting bias. Third, given the substantial time and cost associated with identifying gray literature and non-English studies, and the limited accessibility of non-English sources, these materials were excluded from the review, potentially leading to selection bias. Fourth, most studies included in this review were conducted in Western contexts, with a few originating from Turkey and China. This limited geographic diversity may restrict the generalizability of the findings. Lastly, as this is a scoping review rather than a systematic review, the quality of the included studies was not assessed or used as a basis for exclusion.

## Data Availability

The original contributions presented in the study are included in the article/supplementary material, further inquiries can be directed to the corresponding authors.
